# Prp22 and Spliceosome Components Regulate Chromatin Dynamics in Germ-Line Polyploid Cells

**DOI:** 10.1371/journal.pone.0079048

**Published:** 2013-11-07

**Authors:** Stephen Klusza, Amanda Novak, Shirelle Figueroa, William Palmer, Wu-Min Deng

**Affiliations:** Department of Biological Science, Florida State University, Tallahassee, Florida, United States of America; University of Bern, Switzerland

## Abstract

During *Drosophila* oogenesis, the endopolyploid nuclei of germ-line nurse cells undergo a dramatic shift in morphology as oogenesis progresses; the easily-visible chromosomes are initially polytenic during the early stages of oogenesis before they transiently condense into a distinct ‘5-blob’ configuration, with subsequent dispersal into a diffuse state. Mutations in many genes, with diverse cellular functions, can affect the ability of nurse cells to fully decondense their chromatin, resulting in a ‘5-blob arrest’ phenotype that is maintained throughout the later stages of oogenesis. However, the mechanisms and significance of nurse-cell (NC) chromatin dispersal remain poorly understood. Here, we report that a screen for modifiers of the 5-blob phenotype in the germ line isolated the spliceosomal gene *peanuts*, the *Drosophila* Prp22. We demonstrate that reduction of spliceosomal activity through loss of *peanuts* promotes decondensation defects in NC nuclei during mid-oogenesis. We also show that the Prp38 spliceosomal protein accumulates in the nucleoplasm of nurse cells with impaired *peanuts* function, suggesting that spliceosomal recycling is impaired. Finally, we reveal that loss of additional spliceosomal proteins impairs the full decondensation of NC chromatin during later stages of oogenesis, suggesting that individual spliceosomal subcomplexes modulate expression of the distinct subset of genes that are required for correct morphology in endopolyploid nurse cells.

## Introduction

In the cell, spatiotemporal control of gene expression is crucial for cell-fate determination, maintenance of cell identity, and homeostasis. One of the most important ways in which the cell achieves these aims is through condensation and decondensation of chromosomes at the local and global levels through epigenetic processes. Previous research has uncovered multiple instances in which global chromosome condensation/decondensation is important in developmental contexts. For example, efficient cell migration requires a high level of global chromosome condensation through accumulation of repressive histone marks [1]. Studies have also shown that global decondensation of sperm and oocyte nuclei is an important factor for fertility [2]. Defects in the *microcephalin* (*MCPH1*) gene or its binding partner, *SET, nuclear oncogene*, promote precocious chromosomal condensation, which is thought to be the key defect leading to microencephaly (reduction in human brain size); in cell culture, simultaneous knockdown of condensin II and SET alleviated the precocious chromosomal condensation defect [3]. Last, comparative studies between normal cells and cancerous cells illustrate distinct differences in higher-order spatial organization of chromosomes, implying that defective organization of chromatin may activate ectopic expression of genes that may contribute to tumorigenesis [4]–[8].

During *Drosophila* oogenesis, a series of coordinated events and signal transduction pathways in somatic and germ-line cells prepare the oocyte for maturation and fertilization prior to the onset of embryogenesis. In each *Drosophila* egg chamber, sixteen germ cells (fifteen nurse cells and one oocyte) are encapsulated by an epithelial layer of somatic follicle cells. Whereas both somatic and germ-line cell lineages are instrumental in establishing and maintaining cell polarity in the oocyte [9]–[11], the primary roles of nurse cells are the production and synthesis of RNAs and proteins required for oocyte development, and deposition of maternal reserves needed during early embryogenesis before zygotic transcription is activated [12]. Once cell-fate identity in the germ line is established, prior to the egg chamber budding from the germarium, the nurse cells enter a variant developmental cycle known as the endocycle, in which mitosis is blocked and DNA is endoreplicated once every cycle [13]. Intriguingly, the major chromosomal arms of NC nuclei are easily visualized as a result of endoreplication; in the first four stages of oogenesis, NC nuclei remain in a compact, semi-disordered polytenic form, until activation of condensin II complex disassembles the polytenic chromosomes [14] and promotes condensation of the NC chromatin into a 5-blob state (one blob for each major chromosomal arm – X, 2L, 2R, 3L, and 3R). From stage 6 onwards, the NC chromosomes no longer contain visible structures, instead dispersing into a non-random, diffuse state which is reminiscent of formation of chromosomal territories within the nucleus during mitotic interphase [15].

The mechanism and significance of nurse-cell chromatin dispersal (NCCD) is elusive and poorly understood. NCCD may facilitate an increase in rapid ribosome synthesis [13], because NC nucleoli (the sites of ribosome biogenesis) are found in regions of the nucleoplasm devoid of chromatin. Thus, the nucleolus also displays a dynamic re-distribution contingent on NCCD [16]. Although it is still unknown whether ribosomal activity increases after NCCD, recent studies suggest that failure to disperse NC chromatin does not affect processing of rRNA intermediates required for nucleolar formation [16]. A multitude of genes involved in different processes that affect NCCD, including factors such as pUf68, Squid, and Hrb27C, were also previously implicated in splicing in *Drosophila* [17], [18]. However, in the *Drosophila* germ line, the effects of these proteins on splicing were limited to studies of expression of Ovarian Tumor (Otu), a protein with obscure function that is required for female gamete development and germ-line chromosome organization [19], [20]; unexpectedly, the hnRNP Squid does not affect the alternative splicing of *otu*, but is required for expression of Otu protein. In addition, the hnRNP protein Hrb27C does not affect alternative splicing of the germ-line gene *oskar*, but is required for translational regulation [21]. The alternative splicing factor PUf68 directly affects *otu* splicing in the germ line; however, it has also been implicated in transcriptional regulation of other genes, independent of splicing [22]. Since all three of these genes involved in NCCD are involved in multiple developmental processes, the contribution of spliceosomal activity for NCCD remains obscure.

Here, we report the isolation of the *Drosophila* spliceosomal gene Prp22, also known as *peanuts* (*pea*), from a modifier screen for enhancers/suppressors of the 5-blob phenotype in nurse-cell nuclei. As the *Drosophila* homolog of the yeast Prp22p ATP-dependent DEAH-box helicase, *pea* may also mediate several levels of spliceosomal activity that affect downstream nuclear export of the spliced RNA to the cytosol [23]–[27]. In yeast, defects in Prp22p lead to splicing aberrations through defects in unwinding spliced RNA from the spliceosome, which are ameliorated by mutations in Prp8p that weaken the bonds between the spliceosome [28] and the RNA [26]. To determine whether loss of *pea* function in the *Drosophila* germ line recapitulates chromatin defects seen in loss of splicing factors, we performed mosaic germ-line clonal analysis of a null *pea* mutation in *Drosophila* oogenesis, and observed the presence of NC chromatin defects in *pea*-null NC nuclei. Knockdown of Pea expression in the germ line with RNAi produces similar defect, and mislocalization of the spliceosome in *pea*-null clones suggests that loss of proper spliceosome activity results in NCCD failure. RNAi knockdown of small nuclear ribonucleoprotein complexes (snRNPs) that are part of the spliceosome also produce NC chromatin defects in mid-oogenesis, similar to those seen in later-stage *pea* mosaic germ-line clones. Therefore, we conclude that robust spliceosomal activity is essential for complete decondensation of NC nuclei into a diffuse state.

## Materials and Methods

### Fly Stocks

The following fly stocks were obtained from Bloomington: *y^1^ cv^1^ otu^13^ v^1^ f^1^*/FM3; P{PZ}*Nup154^1501^ cn^1^*/CyO; *P{A92}pea^1^/CyO; ry^506^*; *snRNA:U1:82Eb^KG00155^*/TM3; *Df(2R)Exel7130*; *w^1118^*; P{w[+mC] = UASp-Act5C.T:GFP}3; w*; P{matα4-GAL4-VP16}V2H and w*; {matα4-GAL4-VP16}V37. The Valium20 stocks used were obtained from Harvard and/or Bloomington: y^1^ sc^*^ v^1^; P{TRiP.HMS00274}attP2 (*snRNP-U1-70K*); y^1^ sc^*^ v^1^; P{TRiP.HMS00528}attP2 (*peanuts*); y^1^ sc^*^ v^1^; P{TRiP.HMS00652}attP2 (*Prp19*); y^1^ sc^*^ v^1^; P{TRiP.HMS00442}attP2 (*eIF4AIII*); y^1^ sc^*^ v^1^; P{TRiP.HMS00361}attP2 (*SmD3*); y^1^ sc^*^ v^1^; P{TRiP.HMS00535}attP2 (*U2A*). The hsFLP; FRT42D, hRFP/CyO stock was a kind gift from J Schleede/LM Stevens. The *peanuts* cDNA clone FI05376 was obtained from DGRC, cloned into a pUASp vector tagged with enhanced GFP at the amino terminus, and transformed into flies.

### Generation of mosaic clones by the FLP-FRT technique

Fly crosses and rearing were performed under standard conditions [29]. The FLP-FRT system was used to induce mitotic recombination and produce clones in the ovary through the heat-shock flipase (hsFLP) on the X chromosome. To obtain whole *pea^c89^* germ-line clones, second- and third-instar larvae were heat-shocked for 2 hours on two consecutive days at 37°C; the larvae were then reared at 25°C for eight days, sorted, and moved to fresh vials with yeast for two additional days before dissection [30]. For generation of *pea^c89^* mosaic germ-line clones, the half-clone protocol [31] was modified as follows: second and third-level instar larvae were heat-shocked at 37°C for one hour every day until dissection, in order to circumvent the extreme lethality of the *pea*-null mutation. Clones were marked by the absence of histone-RFP.

### Immunocytochemistry

Immunocytochemistry was performed under standard conditions [32]. The antibodies used were rabbit anti-Staufen at 1∶5000 dilution (Daniel St Johnston), mouse anti-Gurken at 1∶20 dilution (Developmental Hybridoma Studies Bank - University of Iowa, Department of Biology, 028 Biology Building East, Iowa City, Iowa, 52242–1324), mouse anti-Fibrillarin 38F3 at 1∶500 dilution (Abcam, Inc., Cambridge, MA, USA), NSL3 antibody at 1∶500 (kind gift from Asifa Akhtar), and the rabbit anti-Prp38_N antibody at 1∶125 (kind gift from Dr. Nicholas Tapon), diluted in Can Get Signal Immunostain B (Toyobo USA, Inc., 1540 Broadway, Suite 2530 New York, N.Y.10036, USA). Secondary antibodies used were Alexa Fluor 488 and 546 fluorescent-conjugated antibodies; DAPI was used to stain the nuclei of all cells.

### Confocal microscopy and image analysis

Egg chamber images were acquired with a Zeiss LSM 510 confocal and assembled with Photoshop.

### Imprecise excision of *pea* P-element insertion

Homozygous *pea^1^* females were crossed to the *Bc^1^*/Δ2-3, CyO transposase stock, and third-instar larvae were screened for the presence of the Bc marker and discarded. Males with red/white mosaic eyes were then crossed to *Nup154^1501^*/CyO females to create progeny of individual males with rosy background and rosy colored eyes indicating P-element hopout. Then individual males were backcrossed with the *Nup154^1501^*/CyO flies to balance a stock for individual mobilization events and assayed for viability.

### Sequencing of Imprecise *pea* Mutations

Genomic DNA from *pea* mutant lines was isolated using the Invitrogen PureLink Genomic DNA Purification Kit and amplified using *pea* reverse primer 5′-TTGATGCCCAAGTGGTGGTC-3′ and *rcd1* reverse primer 5′-CATTAGCTCTATGACGCCGTC-3′, and sequenced with both primers (except for C89 which was amplified with *pea* reverse primer 5′-CAGTGGGTCTATCCAGTTCT-3′ and *rcd1* reverse primer 5′-CTTGGCGACTACCTCATCAT-3′, and sequenced with *pea* reverse primer 5′-CAATATTAGCTATCTTGCCGGA-3′).

## Results

### Identification of as a spliceosomal protein that affects NC chromatin dynamics

**Figure 1:**
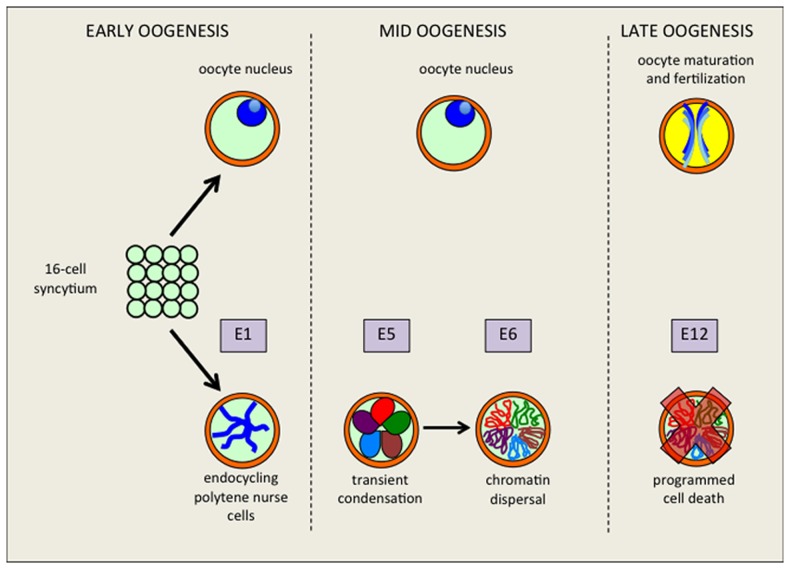
Schematic of NC chromatin states during *Drosophila* oogenesis. (A) Early oogenesis (up to stage 1) marks the formation and budding of the egg chamber from the germarium. The 16-cell syncytium arises from 4 mitotic divisions with incomplete cytokinesis; one cell becomes an oocyte which arrests during meiotic prophase I (with the nucleus condensing to a transcriptionally-quiescent state), while the other fifteen cells abort the meiotic cell cycle and initiate the first endocycle (E1) as polytenic nurse cells. (B) During middle oogenesis (stage 2–10b), NC nuclei facilitate chromosome territory formation by transiently condensing into a ‘5-blob’ configuration during endocycle 5 (E5) and dispersing into a polytene-polyploid state by endocycle 6 (E6) for the remainder of oogenesis. Transient condensation and dispersal occur during stages 4–6 of oogenesis. (C) During late oogenesis (stages 11–14), nurse cells undergo programmed cell death after achieving up to 12 endocycles (E12), dumping their cytoplasmic contents into the oocyte; the oocyte transiently progresses to metaphase I during stage 13, before arrest in preparation for fertilization and egg activation.

During stages 4-6 of oogenesis, the endocycling nurse cells undergo transient condensation of chromatin from a visual polytenic state to a dispersed, polytene-polyploid form (Fig. 1). Previous studies of mutations in genes that affect both NCCD and cell polarity in the oocyte suggested that chromatin dispersal may be required for correct oocyte polarity but could alternatively affect both processes independently of each other. In order to directly test the role of NCCD in oocyte polarity, we used a mutation in *otu* which is haploinsufficient for NCCD [33]. The *otu^13^* mutation confers sterility to females in the homozygous state; in the heterozygous state, females are fertile, but the egg chambers are completely penetrant for NCCD failure and remain stuck in the 5-blob configuration (we refer to this persistent 5-blob configuration as the 5-blob phenotype). We then stained *otu^13^*/+ heterozygous stage-9 egg chambers with Staufen and Gurken antibodies to assay the anteroposterior/dorsoventral (AP/DV) axis polarities of the oocytes (Fig. S1); we did not detect any defects in expression or localization of these polarity determinants. We also successfully rescued the NCCD defects of *otu^13^*/+ heterozygous egg chambers with inclusion of an isoform-specific *otu-104* transgene (Fig. S1E) [33]. Therefore, we conclude that NCCD itself is not crucial for oocyte polarization, and that NCCD defects in *otu^13^*/+ heterozygote egg chambers are solely due to a reduction in *otu* function.

**Figure 2:**
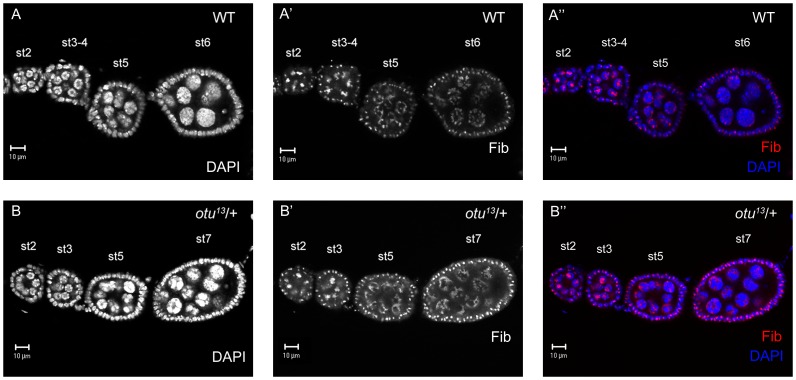
Egg chambers heterozygous for the *otu^13^* mutation fail to disperse NC chromatin. (A-A″) Wild-type ovariole in which polytenic NC nuclei (stages 3–4) transiently condense (stage 5), before dispersing into a diffuse state (stage 6). Fibrillarin (nucleolus marker; red) expands from a compacted form in polytenic NC nuclei to a transient globular phase with transiently-condensed NC chromatin before forming a reticular pattern in dispersed NC nuclei. (B-B″) *otu^13^*/+ egg chambers past stage 6 fail to disperse and retain the globular nucleolus formation until stage 9 of oogenesis, when they accumulate at the distal edges of the NC nuclei.

**Figure 3:**
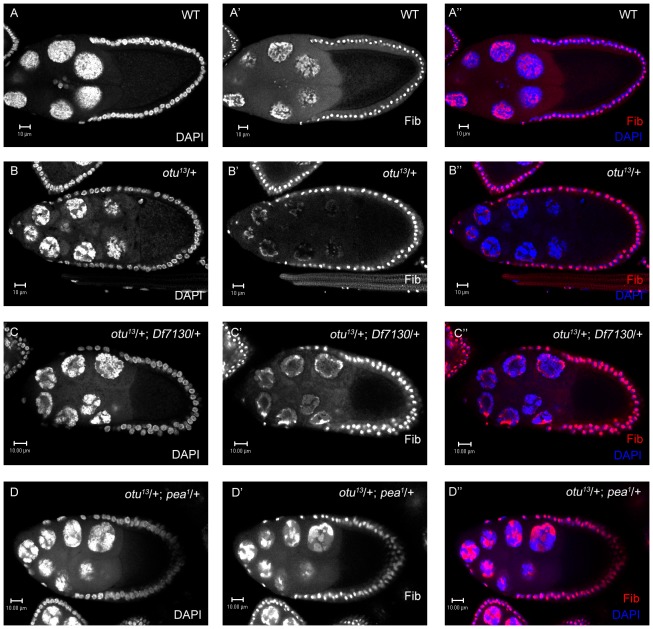
Identification of *pea* as a putative interactor of *otu*. (A-A″) Wild-type stage-9 egg chamber with dispersed NC chromatin and a fully-reticulated nucleoli as marked by fibrillarin (red). (B-B″) *otu^13^*/+ stage-9 egg chambers with NCCD defects and moderately-reticulated nucleoli. (C-C″) In stage-9 *otu^13^*/+; *Df7130*/+ egg chambers (deficiency uncovering *pea*), moderate enhancement of the 5-blob phenotype is seen along with Fibrillarin accumulation at distal edges of NC nuclei. (D-D″) In *otu^13^*/+; *pea^1^*/+ egg chambers, moderate enhancement of the 5-blob configuration plus significant retainment of a globular Fibrillarin pattern is frequently seen.

In an effort to find genes that are required for NCCD, we performed an F1 deficiency screen for modifiers (suppressors/enhancers) of the *otu^13^*/+ 5-blob phenotype, using DAPI to stain the nuclei. By crossing *otu^13^*/+ heterozygous females with deletion stocks uncovering many loci at once, any deletion that allowed NC dispersal (suppression) or hypercondensed NC nuclei formation (enhancer) could identify additional genes required for NCCD. Surprisingly, approximately half of the 208 deletions that we screened exhibited significantly strong enhancement of the *otu^13^*/+ 5-blob phenotype. To determine whether this screen was valid in uncovering new loci involved in NCCD, we focused on a deficiency line known as *Df(2R)Exel7130*, which yields later-stage egg chambers with moderately consistent 5-blob enhancement of NC nuclei. In order to detect subtle differences in NC nuclei which may not be immediately apparent solely by DAPI staining, we also used fibrillarin as a counterstain to visualize the nucleolus (Fig. 2). Fibrillarin is a ribosomal RNA (rRNA) methyltransferase that is located exclusively in the nucleolus [34]; in the wild-type germ line, the nucleolus is concentrated in a small area within a polytenic nucleus (stages 2–4; Fig. 2A′), globular in the 5-blob configuration (stages 4–5; Fig. 2A′), and dispersed along with the NC nuclei (stages 6 and onwards; Fig. 2A′). In *otu^13^*/+ heterozygous egg chambers, the nucleoli and the NC nuclei fail to disperse at stage 6 (Fig. 2B′), with the NC chromatin retaining the 5-blob phenotype. In stage-9 wild-type egg chambers, the nucleoli remains reticulated throughout the NC nuclei during stages 8–10 (Fig 3A′); however, in *otu^13^*/+ stage-9 egg chambers, the nucleoli is overall moderately-reticulated and more embellished on the outer edges of the NC nuclei (Fig. 3B′). In contrast, *otu^13^*/+; *Df(2R)Exel7130*/+ stage-9 egg chambers display enhanced NC chromatin defects and less-reticulated nucleoli (Fig 3C-C″). Based on this result, we performed a secondary screen of available P-element mutations in genes uncovered by the deficiency. We successfully isolated a mutation in an allele of *pea*, *pea^1^*, as an enhancer of the *otu^13^*/+ 5-blob phenotype. *otu^13^*/+; *pea^1^*/+ stage-9 NC nuclei also appear to be more condensed with less-reticulated nucleoli in comparison to *otu^13^*/+ controls (Fig. 3D-D″), suggesting that a lower dosage of *pea* function reverts NC nuclei from a 5-blob configuration to a more polytenic-like state.

**Figure 4:**
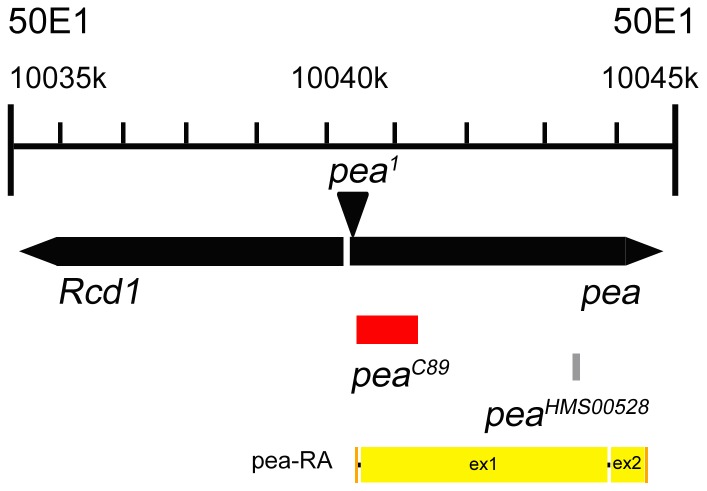
Schematic of the *pea* locus on chromosome 2R. Imprecise excision of the P{A92} P-element (*pea^1^* allele) yielded a 746 bp deletion (red) that removed the transcriptional start site, creating a genetic null mutation (C89). The sequence selected for construction of the *UASp-pea^RNAi^* hairpin is illustrated in grey. For the *pea* transcript (pea-RA), orange boxes mark the 5′ and 3′ UTRs, and yellow boxes mark exons 1 and 2 (ex1 and ex2).

The *pea^1^* allele was first characterized in a screen for P-element-induced mutations that affected spermatogenesis in male flies, in which the P{A92} element was used to hop in randomly across the genome [35]. Since then, no further research has surfaced on the effects of *pea* loss-of-function (LOF) in spermatogenesis; however, because the sequences of *pea* homologs are highly conserved, it is likely that *pea* can perform similar functions in modulating spliceosomal activity in *Drosophila*. The *pea^1^*/CyO stock also contained viable homozygous adults, in which the NC chromatin of mutant egg chambers dispersed normally (data not shown). In previous sequencing data deposited into Flybase, the P{A92} element was reported to be inserted into the 5′-UTR (untranslated region) of *pea* as well as the 5′UTR of a neighboring gene *Rcd1* coding in the opposite direction of *pea*. However, our sequencing reveals that the P{A92} P-element was inserted into the *pea* 5′-UTR 17 base pairs upstream of the *pea* transcriptional start site and did not affect the *Rcd1* 5′-UTR. Imprecise excision of this P-element yielded a single line (*pea^C89^*) with a 746-bp deletion that removes part of the 5′-UTR, the transcriptional start site, and some of the coding sequence of *pea*, creating a genetic null (Fig. 4). The deletion is lethal when homozygous (in contrast to *pea^1^* hypomorphs), so we recombined the *pea^C89^* mutation onto an FRT (Flipase Recognition Target)-containing chromosome to induce mitotic clones in flies heterozygous for the null mutation and assayed the effects of loss-of-*pea* function on cellular processes. In order to confirm *in vivo* that the deletion in the *pea^C89^* allele did not affect Rcd1/NSL3 expression, we created mosaic germ-line clones using a modified version of the heat-shock FLP-FRT protocol [31] which resulted in seven or eight nurse cells being null for *pea* function, while allowing normal Pea expression in wild-type sibling nurse cells. With this procedure, heterozygous nurse cells (hRFP-positive) serve as an internal control for antibody staining of nuclear proteins in NC nuclei without *pea* function. We stained *pea^C89^* mosaic germ-line clones with an antibody against Rcd1/NSL3 [36]. All follicle-cell and germ-line nuclei were positive for Rcd1/NSL3 expression in a wild-type ovariole (Fig. S2A′). In *pea^C89^* mosaic germ-line clones, Rcd1/NSL3 expression remain unchanged in both heterozygous nurse cells (histone-RFP) and *pea^C89^* mutant nurse cells (no histone-RFP; Fig. S2B′, compare arrow to arrowhead), which implies that the NCCD phenotypes seen in the *pea^C89^*-mutant NC nuclei are a consequence of altered spliceosomal dynamics from loss of *pea* function without perturbation of Rcd1/NSL3 expression.

**Figure 5:**
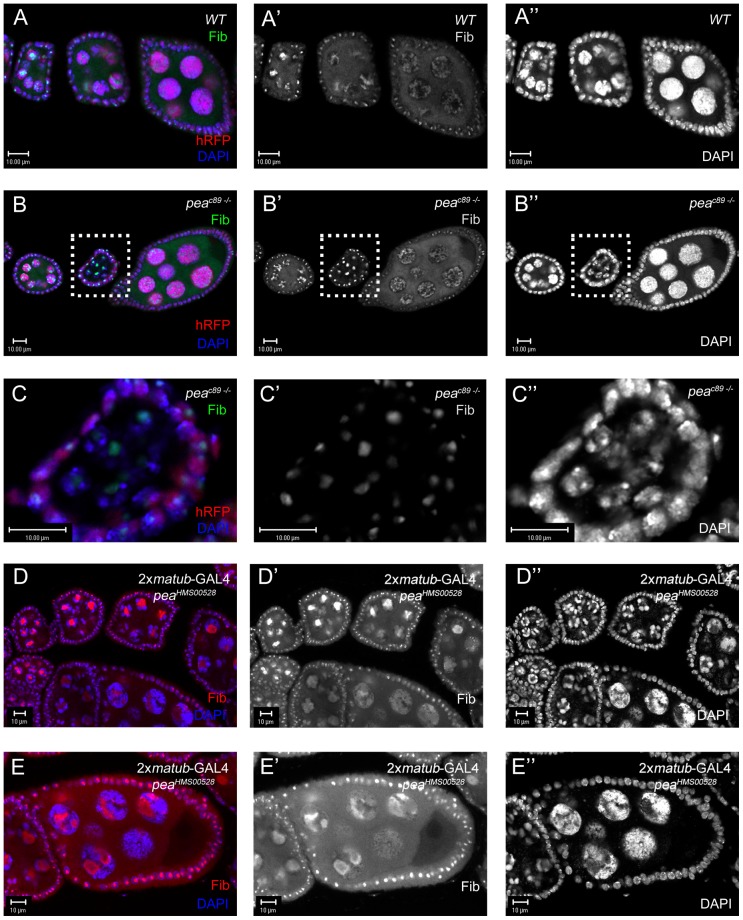
Whole *pea^c89^* germ-line clones arrest at early stages of *Drosophila* oogenesis. (A-A″) In wild-type ovarioles, NC nuclei (DAPI; blue) and the nucleolus (Prp38; green) both undergo a transformation from a polytenic chromatin state (compact nucleolus) into a transient condensed phase (partial dispersal of the nucleolus), before a final dispersed state of both NC chromatin and nucleoli. (B-B″) *pea^c89^* mutant germ-line clones (detected by loss of the histone-RFP marker) invariably arrest at approximately stage 2 of oogenesis, as seen by a severely compact nucleolus (Fibrillarin; green). (C-C″) Closeup of the arrested egg chamber reveals a nucleolus (Fibrillarin; green) surrounded by visible polytenic NC nuclei (DAPI; blue). (D-D″) Knockdown of Pea expression outside of the germarium causes frequent arrest during stages 4-5. (E-E″) Stage-8 *pea*-RNAi egg-chamber with NCCD failure.

Generation of whole germ-line clones with the *pea^C89^* allele is not feasible for studying the effects of *pea* LOF on NCCD because whole germ-line clones (no histone RFP; n = 23) arrest during stages 2-3 of oogenesis (compare Fig. 5A-A′ with Fig. 5B-B′). Closer examination of the whole germ-line clone within the dashed box (Fig. 5C-C′) shows that the NC nuclei are polytenic with an intact nucleolus. In contrast, egg chambers mosaic for *pea* function in the germ line develop further in oogenesis, with a very high penetrance (n = 49/52; 94.2%) of NCCD failure and occasional growth defects from stage 6-10 of oogenesis, in contrast to wild-type egg chambers (n = 3/207; 1.5%). In addition, later-stage mosaic germ-line clones display various novel NC chromatin phenotypes (granular and/or prominent interchromatin spaces), distinct from the transiently-condensed 5-blob phenotype which suggests that *pea* function is required after NCCD to facilitate proper arrangement of the nuclei into chromosomal territories (Fig. S3). We also used RNAi knockdown of *pea* expression in the germ line to determine whether RNAi knockdown caused defects in NC morphologies similar to the genetic null. We drove down *pea* expression by combining the *matub*-GAL4 driver [37] with the *UASp-pea^RNAi^* VALIUM20 line, (in which the *αTub67C* promoter expresses GAL4 maternally after the formation of the 16-cell cyst, but is low or absent in earlier regions of the germarium (Fig. S4). Knockdown of *pea* function with the *matub*-GAL4 drivers frequently arrested egg chambers at stages 4–5 (Fig. 4D-D″), as shown by the lack of globular nucleolus dispersal. Occasionally, *UASp-pea^RNAi^* egg chambers progressed into later stages (Fig. 4E-E″) and failed to disperse their NC chromatin with incompletely-reticulated nucleoli, supporting our conclusion that Pea expression is required before and after egg-chamber formation for NCCD to occur.

**Figure 6:**
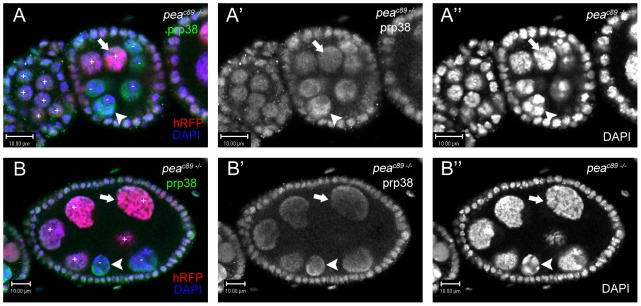
NC nuclei null for *peanuts* function display altered spliceosome localization. (A-A″) Wild-type NC nuclei (marked with histone-RFP, [+])undergoing dispersal display homogenous Prp38 staining, while *pea^c89^* mutant NC nuclei (no histone-RFP, [-]) retain Prp38 localization (green) in interchromatin spaces and a subset of NC chromatin (compare arrow and arrowhead, respectively). Earlier adjacent egg chamber displays wild-type polytene NC nuclei with homogenous Prp38 staining (marked with histone-RFP, [+]). (B-B″) In a stage-7 egg chamber, *pea*-null mutant NC nuclei (arrowhead) remain undispersed and continue to exhibit enhanced Prp38 localization on chromatin in contrast to wild-type dispersed NC nuclei (arrow).

Since other putative homologs of *pea* have been implicated in spliceosomal processes, we stained *pea^C89^* mosaic germ-line clones with an antibody against Prp38 to visualize the levels and localization of the spliceosome within the same egg chamber. Prp38 is an essential spliceosomal protein that is dispensable for spliceosomal assembly but required for catalytic activation of the assembled spliceosome by release of the U4 snRNP [38], [39]. Characterization of *Drosophila* Prp38 indicates a largely conserved function in which Prp38 associates in complex with several components of the U5 snRNP (such as Prp8), the Prp19 complex, and several other splicing factors such as Hrb87F, Hrb98DE, and MFAP1, congruent with its role in activation of the spliceosome [38]. Prp38 expression in a wild-type ovariole is nuclear (Fig. S5A-A″), and transient enhancement of Prp38 staining is occasionally detected between chromosome arms of polytenic and 5-blob NC nuclei (Fig. S5A′; inlay). In order to detect any putative changes in Prp38 expression/localization in NC nuclei with loss of *pea* function, we compared Prp38 staining in hRFP-positive nurse cells (as an internal control) with Prp38 staining of *pea*-null NC nuclei (hRFP-negative). Coincident with NCCD failure, Prp38 localization in *pea^C89^* mosaic germ-line clones is altered, such that the Prp38 signal accumulates largely in between the NC chromatin, although it is also detected on a portion of the chromosomal arms (compare arrow to arrowhead; Fig. 6A-A″). In later-stage egg chambers, Prp38 expression in wild-type NC nuclei remains homogenous while *pea*-null nurse cells retain the 5-blob configuration and altered localization (Fig. 6B-B″). Some irregularities in the shape and morphology of hRFP-positive nurse cells were detected in later-stage mosaic germ-line clones (Fig. 6B and Fig. S2); while we cannot rule out the possibility of *pea*-null NC nuclei affecting the morphology of heterozygous siblings through lack of putative cytoplasmic factors (or other factors such as cellular stress), the persistence of Prp38 altered localization in Fig. 6 demonstrates that spliceosome dynamics are impaired in *pea^C89^* mosaic germ-line clones, which promotes 5-blob dispersal failure.

**Figure 7:**
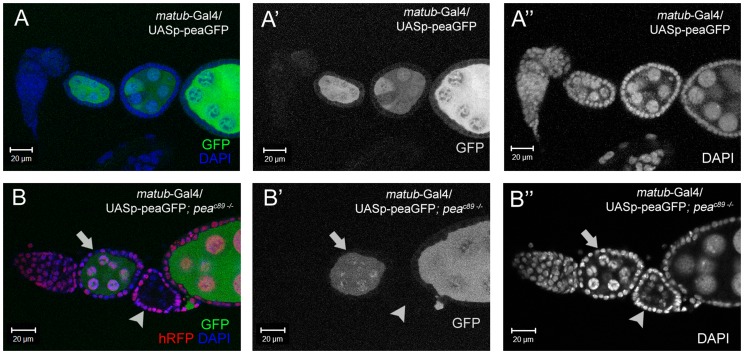
Transgenic overexpression of Pea is silenced in *pea*-null NC nuclei. (A-A″) Ovariole that shows the expression of GFP-tagged Pea in the nuclei and the cytoplasm of the nurse cells. (B-B″) Stage-4 control NC nuclei (hRFP, arrow) expresses PeaGFP strongly. Adjacent *pea^c89^* whole germ-line clone (no hRFP) arrests at an earlier stages and does not express PeaGFP.

Next, we wished to determine whether we could rescue the NC chromatin defects by overexpression of Peanuts in the background of mosaic *pea*-null germ-line clones. Overexpression of a GFP-tagged *pea* transgene with the *matub*-GAL4 driver starts at stage 2 of oogenesis (after budding from the germarium), and is ubiquitous in the cytoplasm and NC nuclei of the female germline throughout oogenesis (Fig. 7A-A″). Surprisingly, we did not observe transgenic Pea-GFP expression in multiple *pea^C89^* whole germ-line clones (n = 10); *pea^C89^* whole germ-line clones are still arrested at stage 3 in ovarioles that are overexpressing PeaGFP in the germline (Fig. 7B-B″; compare arrow with arrowhead). Since the *matub*-GAL4 driver is active from stage 2 of oogenesis and onwards (Fig. S4B-B′), this implies that complete knockdown of *pea* function in NC nuclei in the germarium may induce cell differentiation defects and/or global inhibition of transcription, which then prevents transgene expression. While the inability to perform rescue of *pea^C89^* germ-line clones precludes us from definitively connecting the phenotypes seen to complete loss of *pea* function, the advent of similar phenotypes in RNAi knockdown of *pea* in the germline supports our hypothesis that *pea* is required for proper NC chromatin dynamics and spliceosome activity.

### Different NC chromatin defects are correlated with loss of different spliceosomal proteins

Since knockdown of *pea* expression outside of the germarium results in 5-blob dispersal failure, we tested multiple RNAi lines of other spliceosomal proteins to determine whether 5-blob dispersal failure is a consequence of reduction in general spliceosomal activity. RNAi knockdown of SmD3, a Sm core component assembled into U snRNPs for spliceosomal function [40], [41] with the *matub*-GAL4 driver results in developmental arrest and degeneration of egg chambers by stage 5, similar to Pea knockdown, which provides further evidence that spliceosome function is required for NCCD and oogenesis progression (Fig. S6A-A″). Downregulation of eIF4AIII, a component of the exon-exon junction complex deposited onto spliced RNA [42], produces later-stage egg chambers with NCCD failure (Fig. S6B-B″). The exon-exon junction complex has previously been shown to assemble on spliced mRNA and promote nuclear export; in addition, there is also evidence that the exon-exon junction complex is directly involved in the splicing of heterochromatic transcripts with large introns [43]–[46]. The retention of the 5-blob configuration in eIF4AIII LOF later-stage egg chambers therefore could be a result of defective mRNA export and/or splicing of factors that promote NCCD.

Loss of two different spliceosomal components, Prp19 and U2A, through RNAi knockdown creates distinct NC chromatin defects that differ from the canonical 5-blob phenotype. Prp19 is a putative scaffolding protein for the Nineteen Complex; although it is not found to be a specific component of the spliceosome, it is required for both steps of intron removal in the lariat pathway through spliceosomal conformational changes [47], [48]. After stage 6, the NC nuclei retain some semi-blob characteristics, but change into the ‘bowl’ phenotype from stage 9 onwards, in which the nucleolus does not disperse, but remains clustered in an interchromatin space (reminiscent of *smn^A073^* stage-10 germ-line clones [49]; Fig. S6C-C″), correlating defects in spliceosomal activation with NC chromatin-morphology defects. U2A is a specific component of the U2 snRNP that is essential for viability in *Drosophila* [50]; here, knockdown of U2A creates NC nuclei with a disordered morphology that is distinct from the 5-blob phenotype (Fig. S6D-D″). The U2 snRNP is needed for recruitment to facilitate assembly of the U4, U5, and U6 snRNPs on the RNA prior to spliceosomal catalysis [50]; however, with the presence of multiple other proteins and RNAs in the U2 snRNP, it is possible that knockdown of U2A may only impair U2 snRNP efficiency, allowing only partial NCCD to occur. RNAi knockdown of the U1-snRNP component snRNP-U1-70K with the *matub*-GAL4 driver produces later-stage egg chambers with multiple defects (arrest, apoptosis); NC nuclei in rare stage-9 snRNP-U1-70K RNAi egg chambers fail to disperse, and remain in the 5-blob configuration (Fig. S6E-E″). The snRNP-U1-70K protein is reported to recruit the U1 snRNP to the 5′-splice donor site of mRNA through protein interactions with other splicing factors, and is required for the initiation of ordered spliceosome assembly on pre-mRNA [51]. Similarly, the observation of modest 5-blob defects in later-stage egg chambers of the non-coding U1-snRNP RNA component *snRNA:U1:82Eb^KG00155^* (Fig. S6F-F″) suggests that the U1-snRNP is essential for canonical NCCD during stages 4–6.

## Discussion

The spliceosome is an essential complex in all eukaryotic cells and is required for constitutive and alternative splicing of intron-containing mRNA to produce mature mRNAs for translation into proteins. Extensive genome-wide studies in yeast and human cells demonstrate that, in addition to general defects in splicing, different U-snRNP complexes can affect the splicing of transcripts in distinct ways through participation in additional processes, including alternative splicing and repression of premature cleavage by polyadenylation (PCPA) of pre-mRNAs [52]. In *Drosophila*, spliceosome studies in the past have mostly focused on RNAi knockdown of genes and their effects on splicing and/or the role of the spliceosome in controlling *Sxl* splicing in the sex determination pathways [53], [54]. There has been less emphasis on the developmental consequences of spliceosome defects in the fly beyond sex determination. A previous study using RNAi knockdown in S2 cells identified spliceosomal and non-spliceosomal proteins implicated in alternative splicing of the *adar*, *paralytic*, and *dscam* transcripts (expressed in S2 and neuronal cells, all three genes required for proper neural function and development [55]. In imaginal wing discs, RNAi knockdown of spliceosomal components promotes G2/M cell-cycle arrest and apoptosis, as seen in the reduction of the number of mitotically-active cells assayed through phospho-histone 3 (PH3) staining [55]; another RNAi-knockdown study correlate spliceosomal loss-of-function with shortened spindle defects in the mitotic chromosome [56]. In *Drosophila* oogenesis, examination of defects in the alternative splicing gene *pUf68* connected NCCD failure to a defect in the alternative splicing of the *otu* gene, which is required for germ-line chromatin organization [17]. In addition, defects in the U2-snRNP component *noisette* produce a range of oogenic defects depending on the severity of the allele, including novel abnormal condensation of NC nuclei, delayed development, sensory bristle growth, and fertility [57]. Mutations in SMN also produce NCCD defects in the female germ line, which also suggest that impaired snRNP assembly may be a factor in the NCCD phenotype; the RNA-helicase Gemin 3 also co-localizes with SMN and retains NCCD failure in the mutant germ line as well [58], [59]. Therefore, defects in spliceosome integrity/dynamics can have dramatic effects on multiple aspects of development.

In yeast, Prp22p has been implicated in splicing fidelity through a proofreading function during exon ligation, and release of the U5 snRNP from spliced mRNA [23], [25]–[27], [60]. The *Drosophila* homolog of Prp22p, *pea*, was originally identified in a P-element screen for genes that affect male spermatogenesis [35]. Since then, general studies of the spliceosome have also implicated *pea* in G2/M cell-cycle regulation [38] and alternative splicing of the *dscam* transcript [55]. Here, we show that *pea* function is required for various processes in the germ line. Clonal induction of the *pea^c89^* null mutation in whole germ-line clones invariably causes an early-stage arrest of egg chambers, suggesting that expression of ea–rly oogenesis genes has been compromised, although the NC nuclei are polytenic and the nucleolus appears to be intact (this is further supported by our inability to overexpress transgenic PeaGFP in germlines devoid of all *pea* function). Mosaic germ-line clones allow development of the egg chamber to later stages of oogenesis; however, the mutant nurse cells fail to disperse their chromatin, resulting in a 5-blob phenotype by stage 6, which can often progress to novel morphologies in later stages. RNAi knockdown of *pea* in the germ line with a *nanos*-GAL4 driver results in small ovaries (due to expression in germaria; data not shown); however, the *matub*-GAL4 drivers also allow development of egg chambers up to stage 8 with NC 5-blob defects. From these results, it is clear that *pea* expression is crucial in germ-line stem cells for germ-cell formation, whereas loss of *pea* expression either outside of the germarium or in mosaic germ-line clones (where mitotic recombination is induced in a differentiating germ cell) results in 5-blob dispersal failure in mid-oogenic egg chambers. The stage-dependent differences in *pea* LOF phenotypes likely reflect defects in splicing of factors expressed at distinct times in the germ line, as oogenesis progresses from an undifferentiated state to a committed nurse-cell fate.

Spliceosomal localization in wild-type vs *pea*-null NC nuclei (as assayed with Prp38 antibody) is significantly altered in that during stage 5 of *Drosophila* oogenesis localization of Prp38 antibody is seen in interchromatin spaces, and occasionally appears to localize on a portion of NC chromosomal arms. In wild-type egg chambers, this enrichment of Prp38 is seen in interchromatin spaces in polytenic and transiently-condensed NC nuclei. The persistent altered localization seen in *pea*-null NC nuclei during stages 6-7 of *Drosophila* oogenesis suggests that the spliceosome is unable to disperse from the chromatin/interchromatin spaces, which suggests a loss of spliceosomal reassembly/disassembly at these sites. The observation that the Prp43p helicase is predominantly localized to the nucleolus in yeast cells is intriguing since Prp43p is also closely associated with Prp22p and is required for removal of the intron lariat from the spliced mRNA [61]–[65]. Prp43p is also required for ribosome biogenesis, the processing of pre-rRNA to mature rRNA [61]–[65], which suggests that NCCD could be a result in defects in pre-rRNA splicing. However, examination of *suppressor-of-hairy-wing* (*(Su)Hw*) mutations that fail to disperse NC nuclei did not find any evidence of defects in ribosome biogenesis via mis-processing of rRNA intermediates [16]. A subset of ribosomal proteins has also been shown to interact with Histone 1 (H1) in the nucleus to influence chromatin repression through modulation of the nucleosomes, suggesting that ribosome-independent functions of specific ribosomal proteins could also contribute to NCCD failure [61]. It is currently unknown if the inability to re-localize the spliceosome may be the cause of NCCD failure or a consequence along with failure to disperse the nucleolus and NC chromatin. Further examination of spliceosomal localization in other 5-blob mutants would determine whether altered spliceosomal localization is a general feature of dispersal failure or specific to loss of spliceosome integrity.

Strangely, *pea*-null nurse cells in mosaic germ-line clones do survive for at least a few stages of oogenesis. Since *pea* is implicated in alternative splicing, a defect in the splicing of *otu* isoforms could account for the NC 5-blob defects. However, given the prominent role that *pea* homologs play in unwinding the spliceosome from spliced mRNA and exon ligation, it is surprising that NC nuclei are able to progress. Impairment of *pea* expression through RNAi outside of the germarium clearly shows that *pea* and spliceosome function are required in differentiated egg chambers for progression to later stages of oogenesis and NCCD. Given that high levels of spliceosome activity are evident in germ-line and follicle-cell nuclei at all stages of oogenesis [38], [57], it is surprising that knockdown of some spliceosomal proteins retains chromatin defects that are distinct from uncondensed (polytene) and partially-condensed (5-blob) chromatin in NC nuclei after stage 6 (as seen in mid-stage *noi* mutant egg chambers [57]). Since there is emerging evidence that establishment and maintenance of chromosomal territories is needed for proper spatiotemporal gene expression [66], [67], some spliceosomal proteins have the capacity to influence chromosome territory formation in NC nuclei of mid-oogenic egg chambers either through defective splicing of genes that are active prior to NCCD or spliceosome-independent roles. Therefore, chromosome territory formation may precede a shift in gene expression in NC nuclei, and splicing defects are correlated with defects in gene expression through aberrant organization of higher-order chromatin organization. While the conditions and significance of NCCD are still unknown, the altered localization of the spliceosome in *pea* mutant nurse cells could potentially provide an explanation for chromatin dispersal failure through loss of splicing of key dispersal factors. In regard to human health, spliceosome defects have been implicated in many diseases, including the neurodegenerative disorder spinal muscular atrophy (mutations in SMN) [68], [69], and retinitis pigmentosa [70], a genetic disease in which progressive deterioration of the retina results in blindness. Future detailed studies of spliceosomal function and dynamics in NCCD may aid us in understanding how splicing affects chromatin dynamics and gene expression, and may uncover novel therapeutic targets for gene therapy to reverse the effects of splicing defects on human health.

## Supporting Information

Figure S1 **NCCD failure does not affect oocyte polarization.** (A) Stage-9 wild-type egg chamber with correct Staufen localization at the posterior of the oocyte. (B) Stage-9 *otu^13^*/+ egg chambers with NCCD failure and wild-type Staufen localization. (C) Stage-9 wild-type egg chamber with wild-type Gurken localization at the dorsoventral corner of the oocyte. (D) NCCD failure in stage-9 *otu^13^*/+ egg chambers does not affect the normal pattern of Gurken expression. (E) Rescue of the 5-blob defect of *otu^13^*/+ with the *otu-104* transgene independent of dorsoventral oocyte polarization as seen by normal Gurken localization.
(TIF)

Figure S2 ***pea^c89^*** **egg chambers with mosaic germ-line clones progress further in oogenesis and the clones fail to disperse the NC chromatin; staining with NSL3/Rcd1 antibody reveals that Rcd1 expression is not affected by the deletion in the** ***pea^c89^*** **allele.** (A-A′′′) In wild-type ovarioles, Rcd1/NSL3 expression is detected in all germ-cell and follicle-cell nuclei. (B-B″′) In a stage-6 egg chamber, wild-type NC nuclei (marked by hRFP, arrow) are dispersed while *pea*-null NC nuclei (no hRFP; arrowhead) fail to disperse. In both cases, Rcd1/NSL3 expression is unaffected.
(TIF)

Figure S3 **Late-stage** ***pea^c89-/-^*** **NC nuclei exhibit distinct chromatin morphologies.** (A) Stage-10 egg chamber germ-line mosaic clone in which a smaller *pea*-null NC nucleus (no histone-RFP) fails to disperse in contrast to wild-type NC nuclei (histone-RFP). (A′) A higher focal plane reveals more *pea*-null NC nuclei with multiple chromatin configurations distinct from the 5-blob phenotype.
(TIF)

Figure S4 **Expression of germ-line GAL4 drivers.** (A-A′) The *nanos*-GAL4 driver is active in the germarium, mainly in germ-line stem cells and 16-cell cysts; the extended expression of Act5C-GFP into very young egg chambers is presumably from the stability of the GFP protein. (B-B′) Expression of UASp-Act5C.T:GFP by the *matub*-GAL4 drivers is not detected in the germarium; activation is first detected in budding egg chambers.
(TIF)

Figure S5 **Prp38 is ubiquitous in all nuclei in the** ***Drosophila*** **egg chamber.** (A-A″) Prp38 (active spliceosome marker) is expressed homogenously in all germ-cell and follicle-cell nuclei except for transient embellishment in interchromatin space in transiently-condensed NC nuclei (inlay in A′ show homogenous Prp38 staining with arrow, and transient embellishment of Prp38 with arrowhead).
(TIF)

Figure S6 **RNAi knockdown of spliceosomal components outside of the germarium produces canonical and novel phenotypes in NC nuclei.** (A-A″) Loss of the Sm core protein SmD3 results in arrest at stage 4-5 before degeneration (marked by arrows). (B-B″) Reduction of the exon-exon junction complex component eIF4AIII in later-stage egg chambers correlates with 5-blob dispersal failure. (C-C″) A novel ‘bowl’ phenotype is seen in stage-9 and stage-10 Prp9-deficient egg chambers. (D-D″) Impairment of the U2-snRNP component U2A produces a semi-random granular phenotype distinct from the canonical 5-blob configuration. (E-E″) Loss of snRNP-U1-70K expression in stage-9 egg chambers produce NC nuclei with the classic NCCD phenotype. (F-F″) Egg chambers with reduced U1-snRNA levels also exhibit 5-blob-like defects in NC chromatin nuclei.
(TIF)

## References

[1] Gerlitz G, Bustin M (2010) Efficient cell migration requires global chromatin condensation. J Cell Sci 123: 2207–2217. 20530575 10.1242/jcs.058271 PMC2886743

[2] Agarwal A, Said TM (2003) Role of sperm chromatin abnormalities and DNA damage in male infertility. Hum Reprod Update 9: 331–345. 12926527 10.1093/humupd/dmg027

[3] Leung JW, Leitch A, Wood JL, Shaw-Smith C, Metcalfe K, et al (2011) SET nuclear oncogene associates with microcephalin/MCPH1 and regulates chromosome condensation. J Biol Chem 286: 21393–21400. 21515671 10.1074/jbc.M110.208793 PMC3122199

[4] Guasconi V, Souidi M, Ait-Si-Ali S (2005) Nuclear positioning, gene activity and cancer. Cancer Biol Ther 4: 134–138. 15725725 10.4161/cbt.4.2.1435

[5] Harnicarova A, Kozubek S, Pachernik J, Krejci J, Bartova E (2006) Distinct nuclear arrangement of active and inactive c-myc genes in control and differentiated colon carcinoma cells. Exp Cell Res 312: 4019–4035. 17046748 10.1016/j.yexcr.2006.09.007

[6] Li C, Shi Z, Zhang L, Huang Y, Liu A, et al (2010) Dynamic changes of territories 17 and 18 during EBV-infection of human lymphocytes. Mol Biol Rep 37: 2347–2354. 19685159 10.1007/s11033-009-9740-y

[7] Marella NV, Bhattacharya S, Mukherjee L, Xu J, Berezney R (2009) Cell type specific chromosome territory organization in the interphase nucleus of normal and cancer cells. J Cell Physiol 221: 130–138. 19496171 10.1002/jcp.21836

[8] Murata S, Nakazawa T, Ohno N, Terada N, Iwashina M, et al (2007) Conservation and alteration of chromosome territory arrangements in thyroid carcinoma cell nuclei. Thyroid 17: 489–496. 17614768 10.1089/thy.2006.0328

[9] Horne-Badovinac S, Bilder D (2005) Mass transit: epithelial morphogenesis in the Drosophila egg chamber. Dev Dyn 232: 559–574. 15704134 10.1002/dvdy.20286

[10] Klusza S, Deng WM (2011) At the crossroads of differentiation and proliferation: precise control of cell-cycle changes by multiple signaling pathways in Drosophila follicle cells. Bioessays 33: 124–134. 21154780 10.1002/bies.201000089 PMC3891805

[11] Riechmann V, Ephrussi A (2001) Axis formation during Drosophila oogenesis. Curr Opin Genet Dev 11: 374–383. 11448623 10.1016/s0959-437x(00)00207-0

[12] Ogienko AA, Fedorova SA, Baricheva EM (2007) [Basic aspects of ovarian development in Drosophila melanogaster]. Genetika 43: 1341–1357. 18069338

[13] Dej KJ, Spradling AC (1999) The endocycle controls nurse cell polytene chromosome structure during Drosophila oogenesis. Development 126: 293–303. 9847243 10.1242/dev.126.2.293

[14] Hartl TA, Smith HF, Bosco G (2008) Chromosome alignment and transvection are antagonized by condensin II. Science 322: 1384–1387. 19039137 10.1126/science.1164216

[15] Bauer CR, Hartl TA, Bosco G (2012) Condensin II Promotes the Formation of Chromosome Territories by Inducing Axial Compaction of Polyploid Interphase Chromosomes. PLoS Genet 8: e1002873. 22956908 10.1371/journal.pgen.1002873 PMC3431300

[16] Baxley RM, Soshnev AA, Koryakov DE, Zhimulev IF, Geyer PK (2011) The role of the Suppressor of Hairy-wing insulator protein in Drosophila oogenesis. Dev Biol 356: 398–410. 21651900 10.1016/j.ydbio.2011.05.666 PMC3143288

[17] Goodrich JS, Clouse KN, Schupbach T (2004) Hrb27C, Sqd and Otu cooperatively regulate gurken RNA localization and mediate nurse cell chromosome dispersion in Drosophila oogenesis. Development 131: 1949–1958. 15056611 10.1242/dev.01078

[18] Van Buskirk C, Schupbach T (2002) Half pint regulates alternative splice site selection in Drosophila. Dev Cell 2: 343–353. 11879639 10.1016/s1534-5807(02)00128-4

[19] Glenn LE, Searles LL (2001) Distinct domains mediate the early and late functions of the Drosophila ovarian tumor proteins. Mech Dev 102: 181–191. 11287191 10.1016/s0925-4773(01)00314-8

[20] Koryakov DE, Mal'ceva NI, King RC, Zhimulev IF (2004) Polytene chromosomes from ovarian nurse cells of Drosophila melanogaster otu mutants. Methods Mol Biol 247: 139–161. 14707346 10.1385/1-59259-665-7:139

[21] Huynh JR, Munro TP, Smith-Litiere K, Lepesant JA, St Johnston D (2004) The Drosophila hnRNPA/B homolog, Hrp48, is specifically required for a distinct step in osk mRNA localization. Dev Cell 6: 625–635. 15130488 10.1016/s1534-5807(04)00130-3

[22] Mitchell NC, Johanson TM, Cranna NJ, Er AL, Richardson HE, et al (2010) Hfp inhibits Drosophila myc transcription and cell growth in a TFIIH/Hay-dependent manner. Development 137: 2875–2884. 20667914 10.1242/dev.049585

[23] Mayas RM, Maita H, Staley JP (2006) Exon ligation is proofread by the DExD/H-box ATPase Prp22p. Nat Struct Mol Biol 13: 482–490. 16680161 10.1038/nsmb1093 PMC3729281

[24] McPheeters DS, Schwer B, Muhlenkamp P (2000) Interaction of the yeast DExH-box RNA helicase prp22p with the 3′ splice site during the second step of nuclear pre-mRNA splicing. Nucleic Acids Res 28: 1313–1321. 10684925 10.1093/nar/28.6.1313 PMC111051

[25] Ohno M, Shimura Y (1996) A human RNA helicase-like protein, HRH1, facilitates nuclear export of spliced mRNA by releasing the RNA from the spliceosome. Genes Dev 10: 997–1007. 8608946 10.1101/gad.10.8.997

[26] Schneider S, Campodonico E, Schwer B (2004) Motifs IV and V in the DEAH box splicing factor Prp22 are important for RNA unwinding, and helicase-defective Prp22 mutants are suppressed by Prp8. J Biol Chem 279: 8617–8626. 14688266 10.1074/jbc.M312715200

[27] Schwer B, Gross CH (1998) Prp22, a DExH-box RNA helicase, plays two distinct roles in yeast pre-mRNA splicing. EMBO J 17: 2086–2094. 9524130 10.1093/emboj/17.7.2086 PMC1170553

[28] Schneider S, Schwer B (2001) Functional domains of the yeast splicing factor Prp22p. J Biol Chem 276: 21184–21191. 11283007 10.1074/jbc.M101964200

[29] Poulton JS, Deng WM (2006) Dystroglycan down-regulation links EGF receptor signaling and anterior-posterior polarity formation in the Drosophila oocyte. Proc Natl Acad Sci U S A 103: 12775–12780. 16908845 10.1073/pnas.0603817103 PMC1568923

[30] Deng WM, Ruohola-Baker H (2000) Laminin A is required for follicle cell-oocyte signaling that leads to establishment of the anterior-posterior axis in Drosophila. Curr Biol 10: 683–686. 10837250 10.1016/s0960-9822(00)00514-5

[31] Caceres L, Nilson LA (2005) Production of gurken in the nurse cells is sufficient for axis determination in the Drosophila oocyte. Development 132: 2345–2353. 15829517 10.1242/dev.01820

[32] Deng WM, Althauser C, Ruohola-Baker H (2001) Notch-Delta signaling induces a transition from mitotic cell cycle to endocycle in Drosophila follicle cells. Development 128: 4737–4746. 11731454 10.1242/dev.128.23.4737

[33] Steinhauer WR, Kalfayan LJ (1992) A specific ovarian tumor protein isoform is required for efficient differentiation of germ cells in Drosophila oogenesis. Genes Dev 6: 233–243. 1737618 10.1101/gad.6.2.233

[34] Aris JP, Blobel G (1991) cDNA cloning and sequencing of human fibrillarin, a conserved nucleolar protein recognized by autoimmune antisera. Proc Natl Acad Sci U S A 88: 931–935. 1846968 10.1073/pnas.88.3.931 PMC50928

[35] Castrillon DH, Gonczy P, Alexander S, Rawson R, Eberhart CG, et al (1993) Toward a molecular genetic analysis of spermatogenesis in Drosophila melanogaster: characterization of male-sterile mutants generated by single P element mutagenesis. Genetics 135: 489–505. 8244010 10.1093/genetics/135.2.489 PMC1205651

[36] Raja SJ, Charapitsa I, Conrad T, Vaquerizas JM, Gebhardt P, et al (2010) The nonspecific lethal complex is a transcriptional regulator in Drosophila. Mol Cell 38: 827–841. 20620954 10.1016/j.molcel.2010.05.021

[37] Benton R, St Johnston D (2003) A conserved oligomerization domain in drosophila Bazooka/PAR-3 is important for apical localization and epithelial polarity. Curr Biol 13: 1330–1334. 12906794 10.1016/s0960-9822(03)00508-6

[38] Andersen DS, Tapon N (2008) Drosophila MFAP1 is required for pre-mRNA processing and G2/M progression. J Biol Chem 283: 31256–31267. 18765666 10.1074/jbc.M803512200 PMC2662187

[39] Blanton S, Srinivasan A, Rymond BC (1992) PRP38 encodes a yeast protein required for pre-mRNA splicing and maintenance of stable U6 small nuclear RNA levels. Mol Cell Biol 12: 3939–3947. 1508195 10.1128/mcb.12.9.3939-3947.1992 PMC360275

[40] Gonsalvez GB, Praveen K, Hicks AJ, Tian L, Matera AG (2008) Sm protein methylation is dispensable for snRNP assembly in Drosophila melanogaster. RNA 14: 878–887. 18369183 10.1261/rna.940708 PMC2327358

[41] Gonsalvez GB, Rajendra TK, Tian L, Matera AG (2006) The Sm-protein methyltransferase, dart5, is essential for germ-cell specification and maintenance. Curr Biol 16: 1077–1089. 16753561 10.1016/j.cub.2006.04.037

[42] Chan CC, Dostie J, Diem MD, Feng W, Mann M, et al (2004) eIF4A3 is a novel component of the exon junction complex. RNA 10: 200–209. 14730019 10.1261/rna.5230104 PMC1370532

[43] Ashton-Beaucage D, Therrien M (2011) The exon junction complex: a splicing factor for long intron containing transcripts? Fly (Austin) 5: 224–233. 21478676 10.4161/fly.5.3.15569

[44] Kataoka N, Diem MD, Kim VN, Yong J, Dreyfuss G (2001) Magoh, a human homolog of Drosophila mago nashi protein, is a component of the splicing-dependent exon-exon junction complex. EMBO J 20: 6424–6433. 11707413 10.1093/emboj/20.22.6424 PMC125744

[45] Le Hir H, Gatfield D, Braun IC, Forler D, Izaurralde E (2001) The protein Mago provides a link between splicing and mRNA localization. EMBO Rep 2: 1119–1124. 11743026 10.1093/embo-reports/kve245 PMC1084163

[46] Roignant JY, Treisman JE (2010) Exon junction complex subunits are required to splice Drosophila MAP kinase, a large heterochromatic gene. Cell 143: 238–250. 20946982 10.1016/j.cell.2010.09.036 PMC2955985

[47] Hogg R, McGrail JC, O'Keefe RT (2010) The function of the NineTeen Complex (NTC) in regulating spliceosome conformations and fidelity during pre-mRNA splicing. Biochem Soc Trans 38: 1110–1115. 20659013 10.1042/BST0381110 PMC4234902

[48] Koncz C, Dejong F, Villacorta N, Szakonyi D, Koncz Z (2012) The spliceosome-activating complex: molecular mechanisms underlying the function of a pleiotropic regulator. Front Plant Sci 3: 9. 22639636 10.3389/fpls.2012.00009 PMC3355604

[49] Lee L, Davies SE, Liu JL (2009) The spinal muscular atrophy protein SMN affects Drosophila germline nuclear organization through the U body-P body pathway. Dev Biol 332: 142–155. 19464282 10.1016/j.ydbio.2009.05.553

[50] Nagengast AA, Salz HK (2001) The Drosophila U2 snRNP protein U2A′ has an essential function that is SNF/U2B″ independent. Nucleic Acids Res 29: 3841–3847. 11557816 10.1093/nar/29.18.3841 PMC55907

[51] Salz HK, Mancebo RS, Nagengast AA, Speck O, Psotka M, et al (2004) The Drosophila U1-70K protein is required for viability, but its arginine-rich domain is dispensable. Genetics 168: 2059–2065. 15611175 10.1534/genetics.104.032532 PMC1448718

[52] Kaida D, Berg MG, Younis I, Kasim M, Singh LN, et al (2010) U1 snRNP protects pre-mRNAs from premature cleavage and polyadenylation. Nature 468: 664–668. 20881964 10.1038/nature09479 PMC2996489

[53] Nagengast AA, Stitzinger SM, Tseng CH, Mount SM, Salz HK (2003) Sex-lethal splicing autoregulation in vivo: interactions between SEX-LETHAL, the U1 snRNP and U2AF underlie male exon skipping. Development 130: 463–471. 12490553 10.1242/dev.00274

[54] Salz HK, Flickinger TW (1996) Both loss-of-function and gain-of-function mutations in snf define a role for snRNP proteins in regulating Sex-lethal pre-mRNA splicing in Drosophila development. Genetics 144: 95–108. 8878676 10.1093/genetics/144.1.95 PMC1207521

[55] Park JW, Parisky K, Celotto AM, Reenan RA, Graveley BR (2004) Identification of alternative splicing regulators by RNA interference in Drosophila. Proc Natl Acad Sci U S A 101: 15974–15979. 15492211 10.1073/pnas.0407004101 PMC528766

[56] Goshima G, Wollman R, Goodwin SS, Zhang N, Scholey JM, et al (2007) Genes required for mitotic spindle assembly in Drosophila S2 cells. Science 316: 417–421. 17412918 10.1126/science.1141314 PMC2837481

[57] Meyer V, Oliver B, Pauli D (1998) Multiple developmental requirements of noisette, the Drosophila homolog of the U2 snRNP-associated polypeptide SP3a60. Mol Cell Biol 18: 1835–1843. 9528755 10.1128/mcb.18.4.1835 PMC121413

[58] Cauchi RJ, Sanchez-Pulido L, Liu JL (2010) Drosophila SMN complex proteins Gemin2, Gemin3, and Gemin5 are components of U bodies. Exp Cell Res 316: 2354–2364. 20452345 10.1016/j.yexcr.2010.05.001

[59] Cauchi RJ (2012) Conserved requirement for DEAD-box RNA helicase Gemin3 in Drosophila oogenesis. BMC Res Notes 5: 120. 22361416 10.1186/1756-0500-5-120 PMC3392723

[60] Ono Y, Ohno M, Shimura Y (1994) Identification of a putative RNA helicase (HRH1), a human homolog of yeast Prp22. Mol Cell Biol 14: 7611–7620. 7935475 10.1128/mcb.14.11.7611-7620.1994 PMC359297

[61] Mayas RM, Maita H, Semlow DR, Staley JP (2010) Spliceosome discards intermediates via the DEAH box ATPase Prp43p. Proc Natl Acad Sci U S A 107: 10020–10025. 20463285 10.1073/pnas.0906022107 PMC2890470

[62] Martin A, Schneider S, Schwer B (2002) Prp43 is an essential RNA-dependent ATPase required for release of lariat-intron from the spliceosome. J Biol Chem 277: 17743–17750. 11886864 10.1074/jbc.M200762200

[63] Leeds NB, Small EC, Hiley SL, Hughes TR, Staley JP (2006) The splicing factor Prp43p, a DEAH box ATPase, functions in ribosome biogenesis. Mol Cell Biol 26: 513–522. 16382143 10.1128/MCB.26.2.513-522.2006 PMC1346897

[64] Combs DJ, Nagel RJ, Ares M Jr, Stevens SW (2006) Prp43p is a DEAH-box spliceosome disassembly factor essential for ribosome biogenesis. Mol Cell Biol 26: 523–534. 16382144 10.1128/MCB.26.2.523-534.2006 PMC1346896

[65] Arenas JE, Abelson JN (1997) Prp43: An RNA helicase-like factor involved in spliceosome disassembly. Proc Natl Acad Sci U S A 94: 11798–11802. 9342317 10.1073/pnas.94.22.11798 PMC23592

[66] Mewborn SK, Puckelwartz MJ, Abuisneineh F, Fahrenbach JP, Zhang Y, et al (2010) Altered chromosomal positioning, compaction, and gene expression with a lamin A/C gene mutation. PLoS One 5: e14342 doi:10.1371/journal.pone.0014342 21179469 10.1371/journal.pone.0014342 PMC3001866

[67] Heard E, Bickmore W (2007) The ins and outs of gene regulation and chromosome territory organisation. Curr Opin Cell Biol 19: 311–316. 17467967 10.1016/j.ceb.2007.04.016

[68] Clelland AK, Bales AB, Sleeman JE (2012) Changes in intranuclear mobility of mature snRNPs provide a mechanism for splicing defects in spinal muscular atrophy. J Cell Sci 125: 2626–2637. 22393244 10.1242/jcs.096867 PMC3403233

[69] Lorson CL, Rindt H, Shababi M (2010) Spinal muscular atrophy: mechanisms and therapeutic strategies. Hum Mol Genet 19: R111–118. 20392710 10.1093/hmg/ddq147 PMC2875050

[70] Grainger RJ, Beggs JD (2005) Prp8 protein: at the heart of the spliceosome. RNA 11: 533–557. 15840809 10.1261/rna.2220705 PMC1370742

