# Supplementary figures and images for: Prp22 and Spliceosome Components Regulate Chromatin Dynamics in Germ-Line Polyploid Cells
Source: PLoS One. 2013 Nov 7;8(11):e79048. doi: 10.1371/journal.pone.0079048 (PMC3820692; doi:10.1371/journal.pone.0079048)

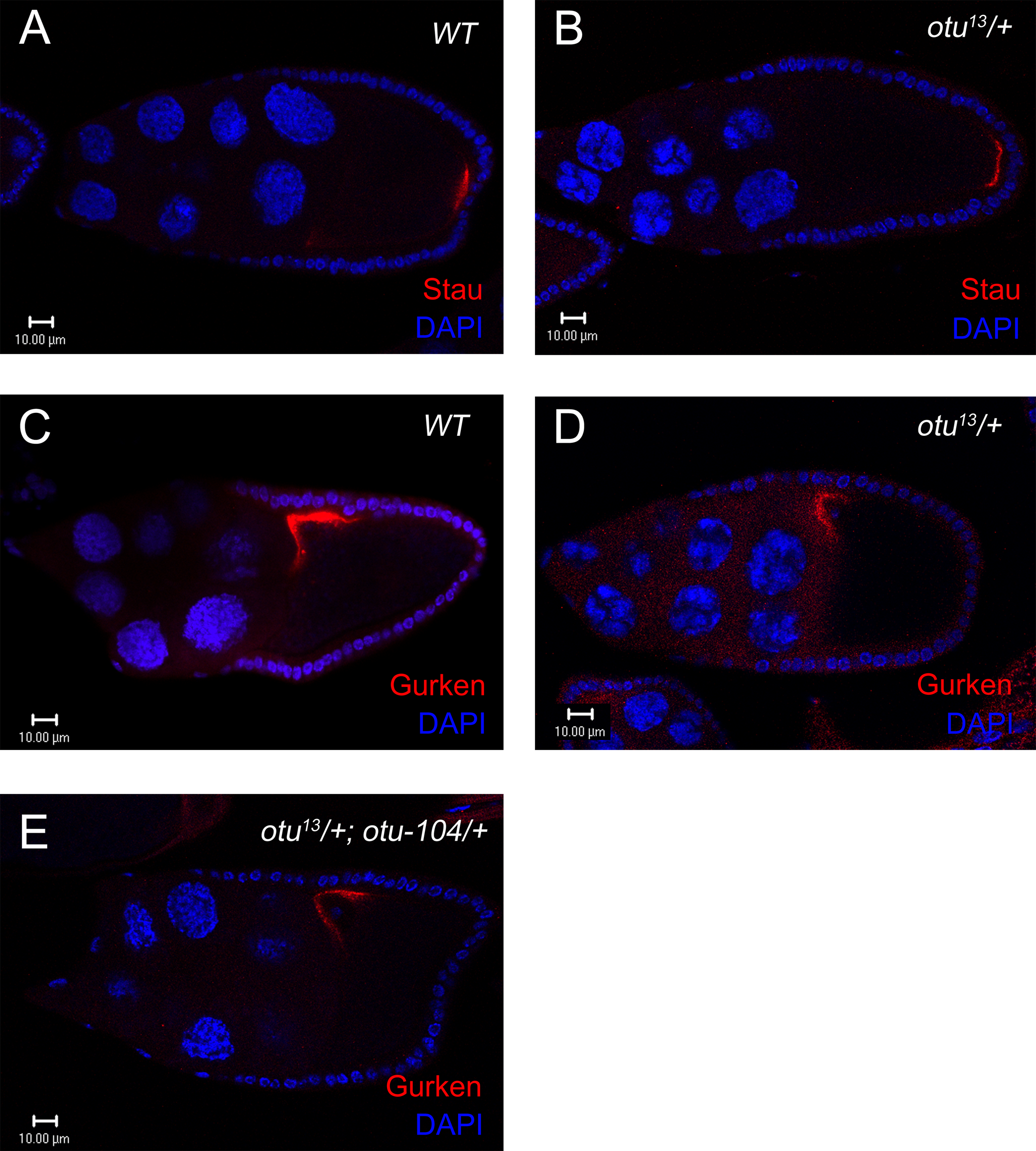

Supplement: Figure S1 — NCCD failure does not affect oocyte polarization. (A) Stage-9 wild-type egg chamber with correct Staufen localization at the posterior of the oocyte. (B) Stage-9 otu13/+ egg chambers with NCCD failure and wild-type Staufen localization. (C) Stage-9 wild-type egg chamber with wild-type Gurken localization at the dorsoventral corner of the oocyte. (D) NCCD failure in stage-9 otu13/+ egg chambers does not affect the normal pattern of Gurken expression. (E) Rescue of the 5-blob defect of otu13/+ with the otu-104 transgene independent of dorsoventral oocyte polarization as seen by normal Gurken localization. (TIF) [file pone.0079048.s001.tif]

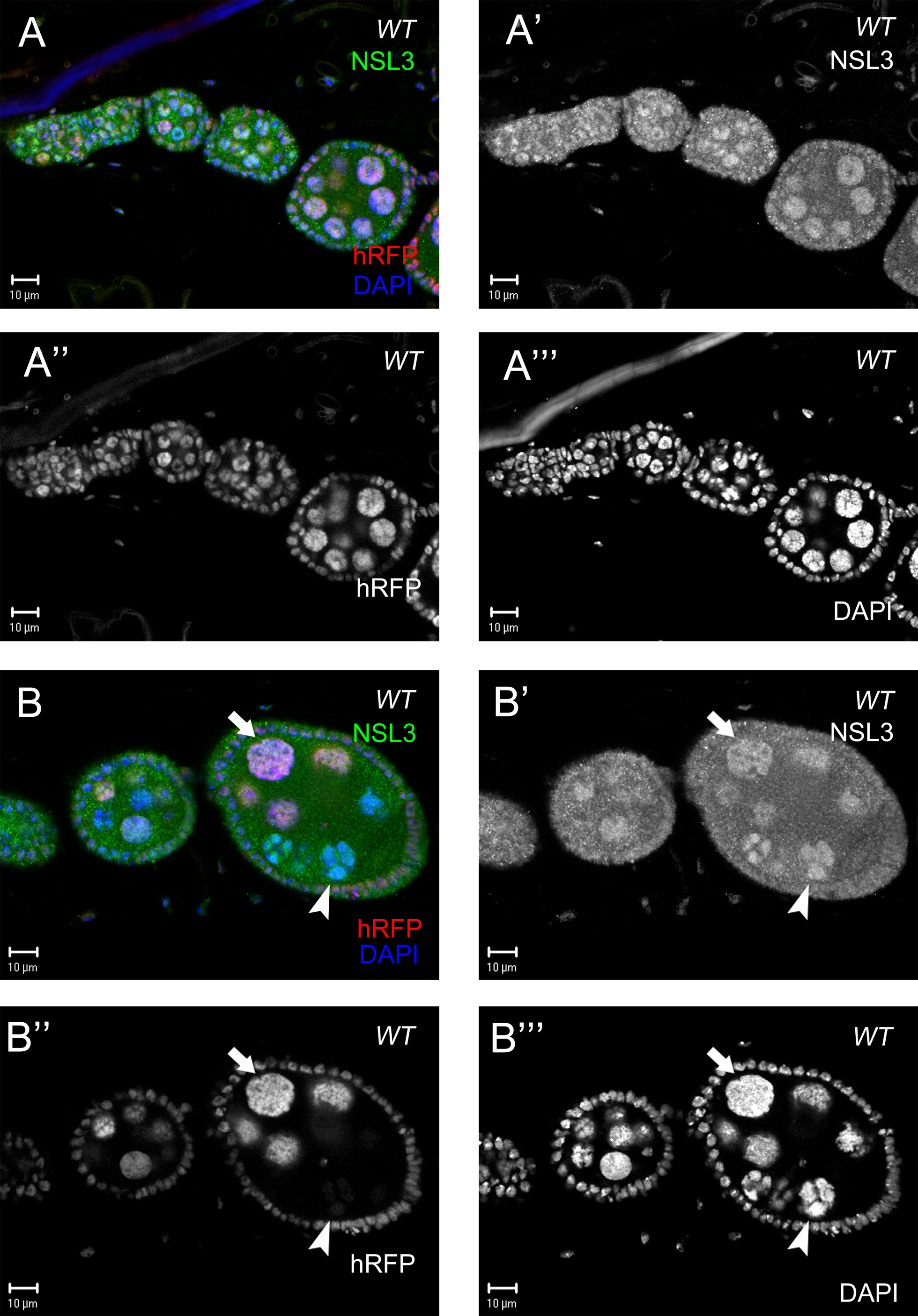

Supplement: Figure S2 — peac89 egg chambers with mosaic germ-line clones progress further in oogenesis and the clones fail to disperse the NC chromatin; staining with NSL3/Rcd1 antibody reveals that Rcd1 expression is not affected by the deletion in the peac89 allele. (A-A′′′) In wild-type ovarioles, Rcd1/NSL3 expression is detected in all germ-cell and follicle-cell nuclei. (B-B″′) In a stage-6 egg chamber, wild-type NC nuclei (marked by hRFP, arrow) are dispersed while pea-null NC nuclei (no hRFP; arrowhead) fail to disperse. In both cases, Rcd1/NSL3 expression is unaffected. (TIF) [file pone.0079048.s002.tif]

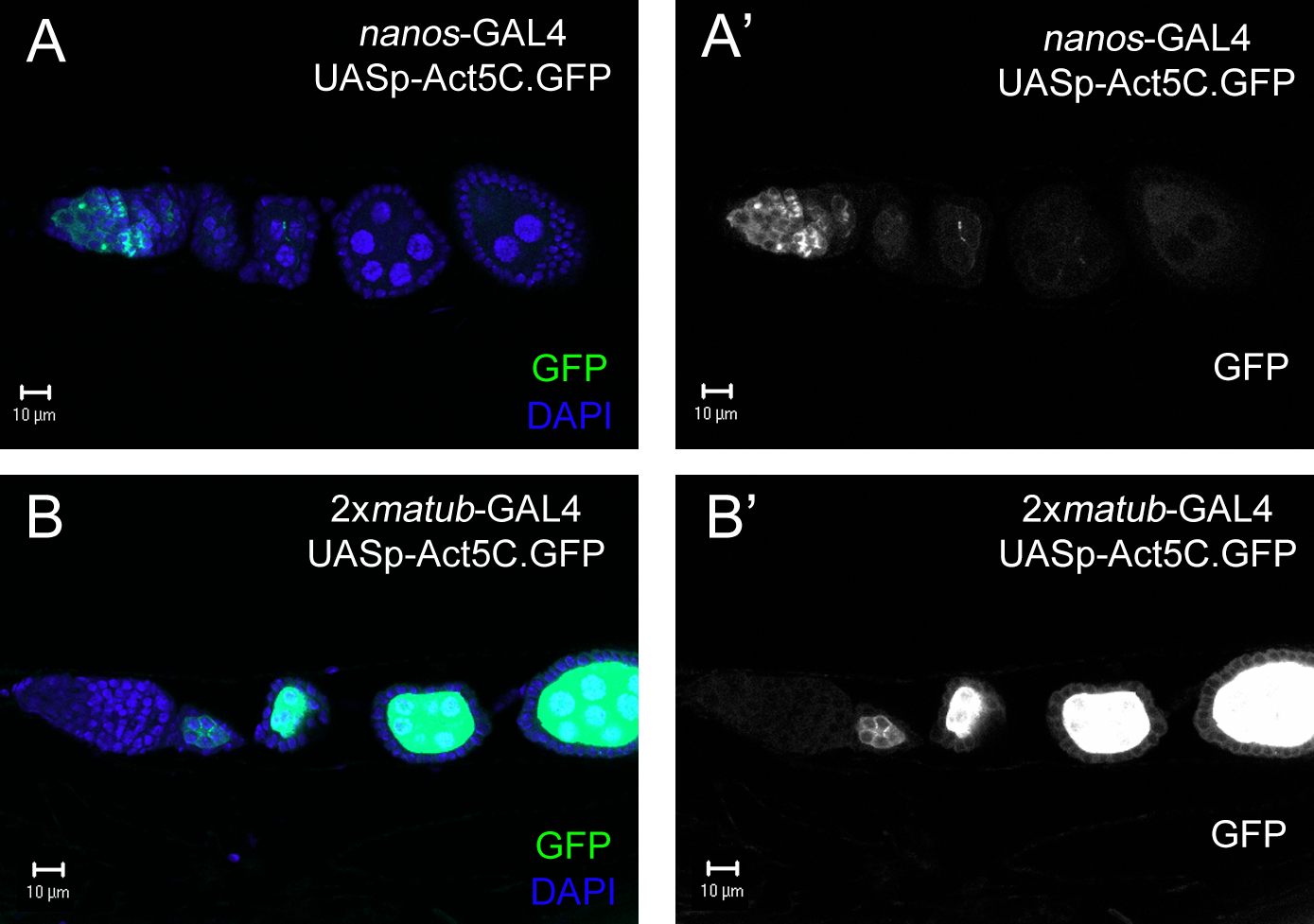

Supplement: Figure S3 — Late-stage peac89-/- NC nuclei exhibit distinct chromatin morphologies. (A) Stage-10 egg chamber germ-line mosaic clone in which a smaller pea-null NC nucleus (no histone-RFP) fails to disperse in contrast to wild-type NC nuclei (histone-RFP). (A′) A higher focal plane reveals more pea-null NC nuclei with multiple chromatin configurations distinct from the 5-blob phenotype. (TIF) [file pone.0079048.s003.tif]

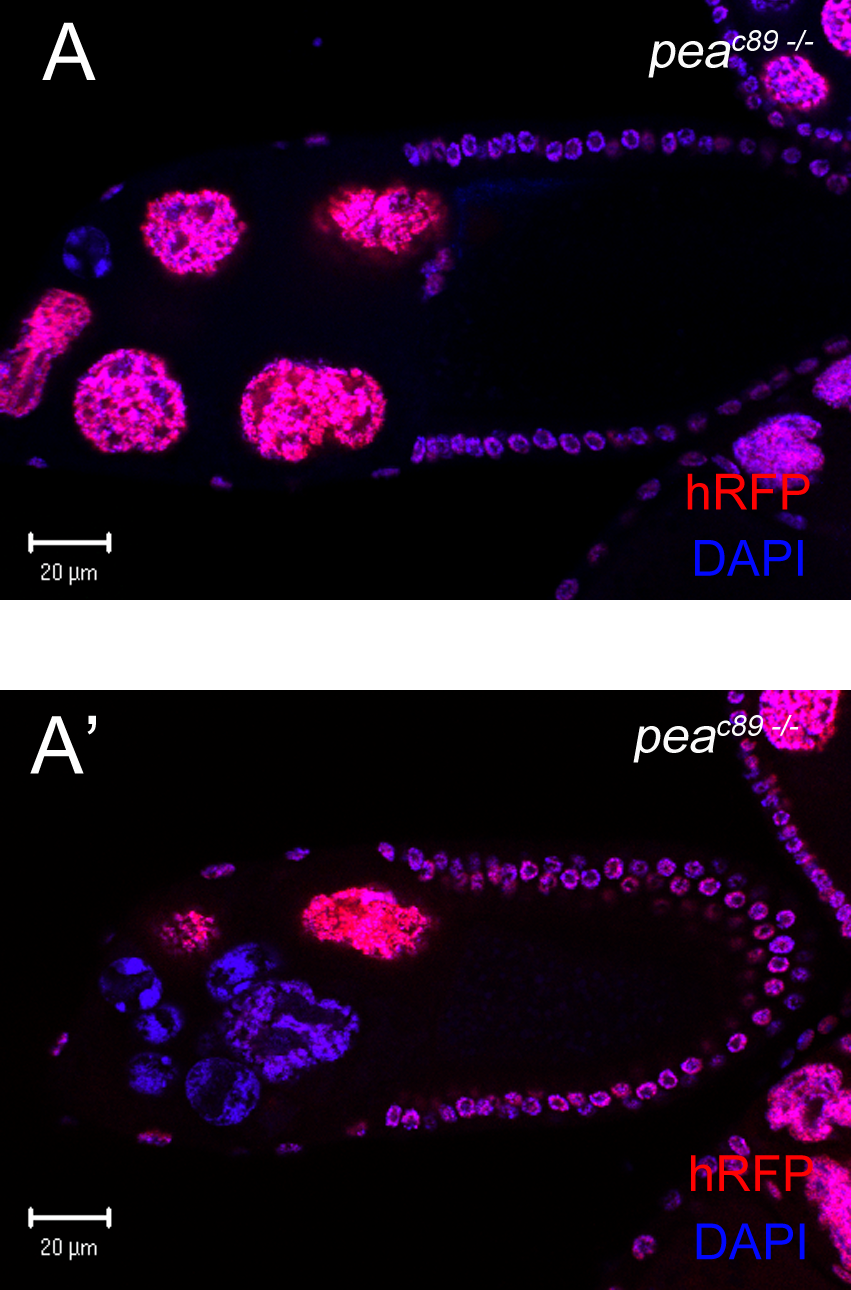

Supplement: Figure S4 — Expression of germ-line GAL4 drivers. (A-A′) The nanos-GAL4 driver is active in the germarium, mainly in germ-line stem cells and 16-cell cysts; the extended expression of Act5C-GFP into very young egg chambers is presumably from the stability of the GFP protein. (B-B′) Expression of UASp-Act5C.T:GFP by the matub-GAL4 drivers is not detected in the germarium; activation is first detected in budding egg chambers. (TIF) [file pone.0079048.s004.tif]

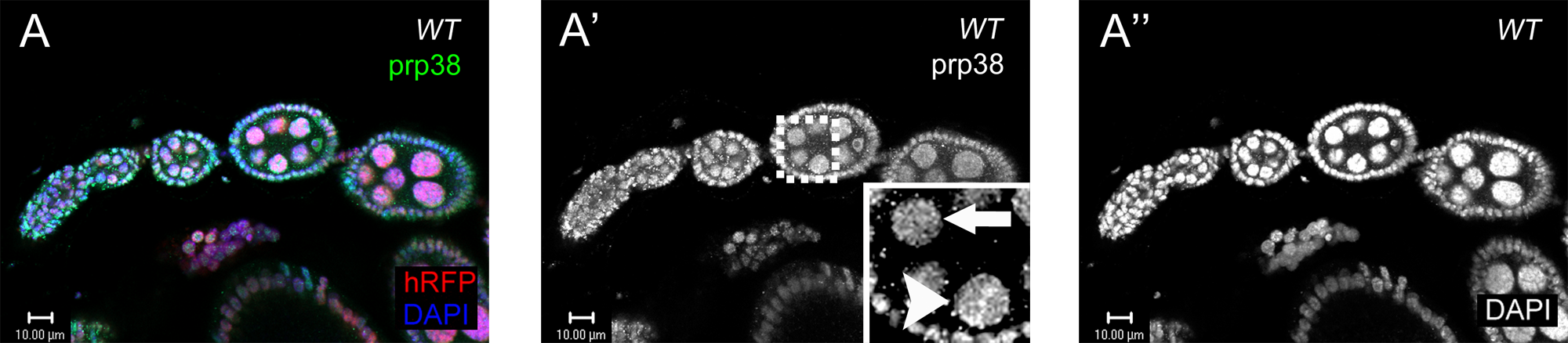

Supplement: Figure S5 — Prp38 is ubiquitous in all nuclei in the Drosophila egg chamber. (A-A″) Prp38 (active spliceosome marker) is expressed homogenously in all germ-cell and follicle-cell nuclei except for transient embellishment in interchromatin space in transiently-condensed NC nuclei (inlay in A′ show homogenous Prp38 staining with arrow, and transient embellishment of Prp38 with arrowhead). (TIF) [file pone.0079048.s005.tif]

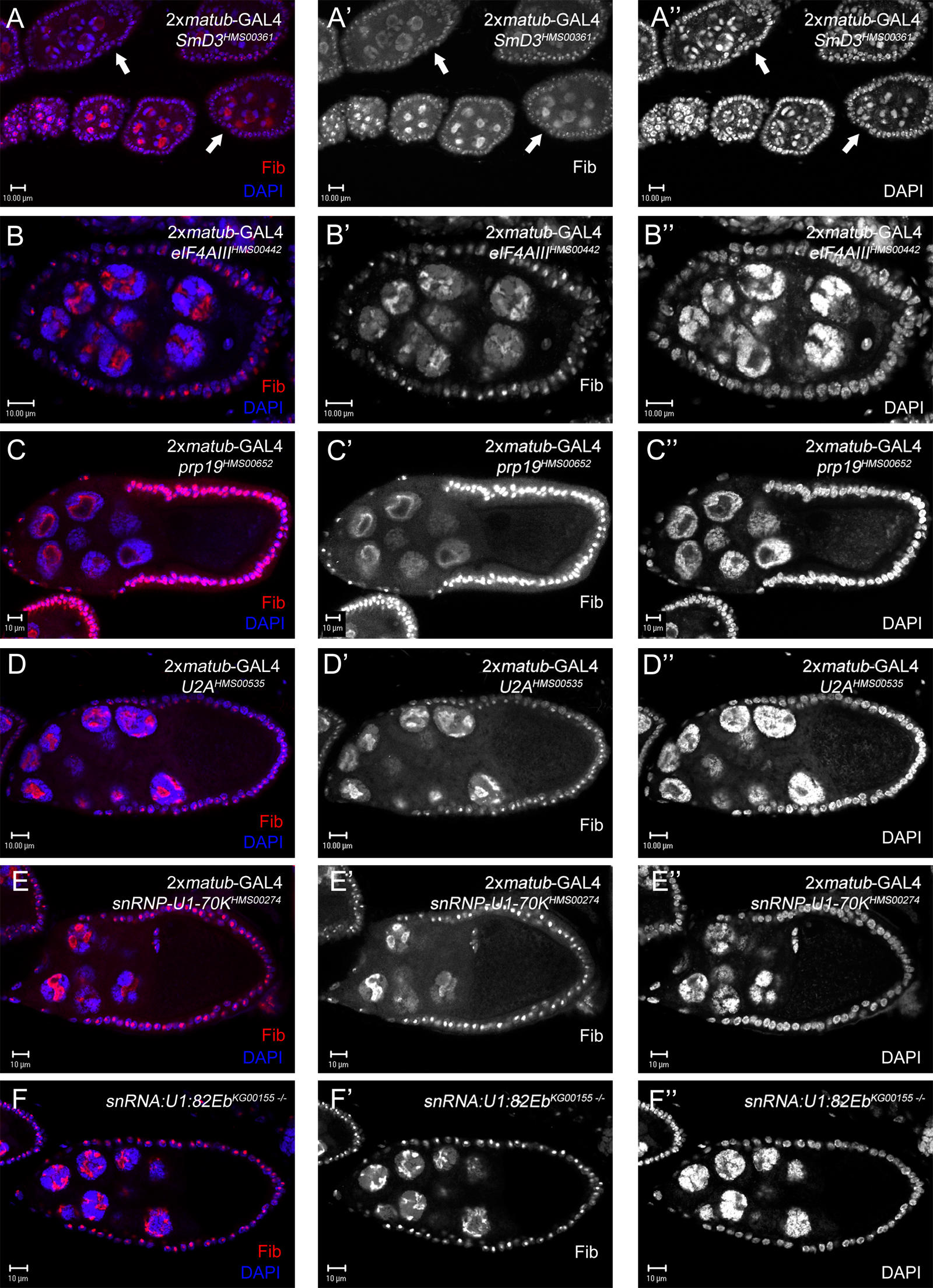

Supplement: Figure S6 — RNAi knockdown of spliceosomal components outside of the germarium produces canonical and novel phenotypes in NC nuclei. (A-A″) Loss of the Sm core protein SmD3 results in arrest at stage 4-5 before degeneration (marked by arrows). (B-B″) Reduction of the exon-exon junction complex component eIF4AIII in later-stage egg chambers correlates with 5-blob dispersal failure. (C-C″) A novel ‘bowl’ phenotype is seen in stage-9 and stage-10 Prp9-deficient egg chambers. (D-D″) Impairment of the U2-snRNP component U2A produces a semi-random granular phenotype distinct from the canonical 5-blob configuration. (E-E″) Loss of snRNP-U1-70K expression in stage-9 egg chambers produce NC nuclei with the classic NCCD phenotype. (F-F″) Egg chambers with reduced U1-snRNA levels also exhibit 5-blob-like defects in NC chromatin nuclei. (TIF) [file pone.0079048.s006.tif]
